# The Combination Treatment of Fosmanogepix and Liposomal Amphotericin B Is Superior to Monotherapy in Treating Experimental Invasive Mold Infections

**DOI:** 10.1128/aac.00380-22

**Published:** 2022-06-07

**Authors:** Teclegiorgis Gebremariam, Yiyou Gu, Sondus Alkhazraji, Eman Youssef, Karen Joy Shaw, Ashraf S. Ibrahim

**Affiliations:** a The Lundquist Institute at Harbor-UCLA Medical Center, Torrance, California, USA; b Beni-Suef University, Beni-Suef, Egypt; c Hearts Consulting Group, LLC, Poway, California, USA; d David Geffen School of Medicine at UCLA, Los Angeles, California, USA

**Keywords:** APX001, APX001A, Gwt1, antifungal, aspergillosis, mucormycosis, fusariosis infection model, IPA, 1-aminobenzotriazole, manogepix, fosmanogepix, fusariosis, infection model

## Abstract

Invasive pulmonary aspergillosis (IPA), invasive mucormycosis (IM), and invasive fusariosis (IF) are associated with high mortality and morbidity. Fosmanogepix (FMGX) is a first-in-class antifungal in clinical development with demonstrated broad-spectrum activity in animal models of infections. We sought to evaluate the benefit of combination therapy of FMGX plus liposomal amphotericin B (L-AMB) in severe delayed-treatment models of murine IPA, IM, and IF. While FMGX was equally as effective as L-AMB in prolonging the survival of mice infected with IPA, IM, or IF, combination therapy was superior to monotherapy in all three models. These findings were validated by greater reductions in the tissue fungal burdens (determined by quantitative PCR) of target organs in all three models versus the burdens in infected vehicle-treated (placebo) or monotherapy-treated mice. In general, histopathological examination of target organs corroborated the findings for fungal tissue burdens among all treatment arms. Our results show that treatment with the combination of FMGX plus L-AMB demonstrated high survival rates and fungal burden reductions in severe animal models of invasive mold infections, at drug exposures in mice similar to those achieved clinically. These encouraging results warrant further investigation of the FMGX–plus–L-AMB combination treatment for severely ill patients with IPA, IM, and IF.

## INTRODUCTION

Invasive pulmonary aspergillosis (IPA), invasive mucormycosis (IM), and invasive fusariosis (IF) are associated with high morbidity and mortality rates that frequently exceed 50%, despite the use of recommended antifungal therapy (1–3). Consequently, treatment with a combination of several antifungal drugs is often considered. This approach is usually supported by growing evidence from *in vitro* studies, animal models of infection, and case reports of clinical experience, since comparative clinical trials are hard to perform for these rare infections.

Fosmanogepix (FMGX) is a first-in-class antifungal prodrug which has been shown to have broad-spectrum *in vitro* activity against several pathogenic fungi, including Candida species, Cryptococcus species, Aspergillus species, Mucorales, Fusarium species, and Scedosporium species (4–8). The active moiety, manogepix (MGX), inhibits the synthesis of glycosylphosphatidylinositol (GPI)-anchored proteins by targeting the conserved fungal Gwt1 enzyme (9, 10). Studies utilizing murine or rabbit models of infection demonstrated potent activity of FMGX against Candida species, including Candida auris (11–13) and Candida neoformans (14), as well as Coccidioides (15), IPA (16), IM (17), Fusarium solani, and Scedosporium (18), and in many cases, this activity was equal to that of the standard-of-care antifungal drugs. Specifically, we recently reported on activity of FMGX that was comparable to that of isavuconazole in treating murine mucormycosis (17). Similarly, FMGX was found to be equally as effective as posaconazole in treating IPA of immunosuppressed mice (16). Finally, FMGX was as effective as a high dose of liposomal amphotericin B (L-AMB) in treating immunosuppressed mice infected with hematogenously disseminated fusariosis or pulmonary scedosporiosis (18).

Several of the mouse models utilized 1-aminobenzotriazole (ABT), a nonselective suicide inhibitor of cytochrome P450 (CYP) enzymes (19), to increase the exposure and half-life of MGX in mice (14, 20). We found that the administration of 50 mg/kg of body weight of ABT 2 h prior to treatment with FMGX extended the MGX serum half-life and area under the concentration-time curve (AUC) by ~9-fold and 18-fold, respectively (16). Thus, the use of ABT in mice allows the resulting exposures to more closely mimic those obtained in humans in phase 1 clinical studies of healthy volunteers (11, 21, 22). Similarly, exposures of AMB in mice are similar to those achieved clinically, with a dose of 5 mg/kg of L-AMB achieving a maximum plasma level of 50 mg/L in mice, which was similar to a 4 mg/kg dose in humans with a peak plasma concentration of ~46 mg/L (23, 24).

Given the severity of IPA, IM, and IF in the immunocompromised patient population, we sought to evaluate the benefit of combination therapy of FMGX plus L-AMB (FMGX+L-AMB) compared to monotherapy by using the immunosuppressed-mouse models of these infections. Our endpoint of efficacy included comparative analyses of survival, measurements of tissue fungal burdens of target organs using quantitative PCR (qPCR) as assessed by Log_10_ conidial equivalents per gram, and histological improvements.

(A portion of this work was presented online at the 31st European Congress of Clinical Microbiology & Infectious Diseases [25].)

## RESULTS

### Antifungal susceptibility.

The MIC values for amphotericin B (AMB) and minimum effective concentration (MEC) values for MGX for the 3 strains used in the infection models are shown in Table 1. A. fumigatus strain AF293 and F. solani strain 95-2478 each had a low MGX MEC of 0.03 μg/mL, whereas R. arrhizus var. *delemar* strain 99-880 had an MGX MEC of 0.25 μg/mL. For AMB, A. fumigatus AF293 and R. arrhizus var. *delemar* 99-880 each had a MIC of 0.25 μg/mL, while F. solani 95-2478 was slightly more resistant, with the MIC registering at 4.0 μg/mL (Table 1). Previous synergy measurement by checkerboard analysis of 18 strains of A. fumigatus and 4 strains of A. flavus has shown that the combination of MGX and AMB is indifferent (26).

**TABLE 1:** Antifungal susceptibilities

Clinical isolate	Value (μg/mL) for:	
	MGX MEC	AMB MIC
Aspergillus fumigatus AF293	0.03	0.25
Rhizopus arrhizus var. *delemar* strain 99-880	0.25	0.25
Fusarium solani 95-2478	0.03	4.0

### Combination therapy of FMGX and L-AMB is superior to monotherapy in treating murine IPA.

The efficacies of monotherapies of FMGX (78 mg/kg) and L-AMB (5 mg/kg) and of the combination of FMGX and L-AMB were evaluated versus the outcomes for the vehicle control (placebo) in a severe delayed-treatment IPA model, where dosing was initiated 48 h postinfection. A dose of 50 mg/kg ABT plus 78 mg/kg FMGX was chosen since previous studies demonstrated that the resulting exposures in mice mimicked those obtained in humans in phase 1 clinical studies of healthy volunteers (11, 21, 22). Similarly, a dose of 5 mg/kg of L-AMB in mice gives rise to a peak plasma concentration close to that resulting from a 4-mg/kg dose in humans (23, 24).

In a separate experiment, the effect of the combination of drugs on the exposure (AUC from dosing to the time of the last measured concentration [AUC_last_]) was assessed in mice. A dose of 10 mg/kg L-AMB was administered alone, with 50 mg/kg ABT, with 26 mg/kg FMGX, and with FMGX+ABT. A dose of 26 mg/kg FMGX was administered with and without 50 mg/kg ABT. There was no difference in the AMB AUC_last_ values without or with ABT (1.37 and 1.38 mg · h/mL, respectively), whereas as anticipated, a large (19-fold) increase in AUC_last_ was observed for MGX when ABT was present. The AMB AUC_last_ value in the presence of both ABT and MGX was 14% higher than the AMB value when dosed with ABT but without MGX (1.60 versus 1.38 mg · h/mL). The MGX AUC_last_ value in the presence of both ABT and L-AMB was 17% lower than the value when MGX+ABT was administered without L-AMB (60.1 versus 72.2 μg · h/mL). These results suggest that there are limited changes in the drug exposures observed when given in combination.

**FIG 1:**
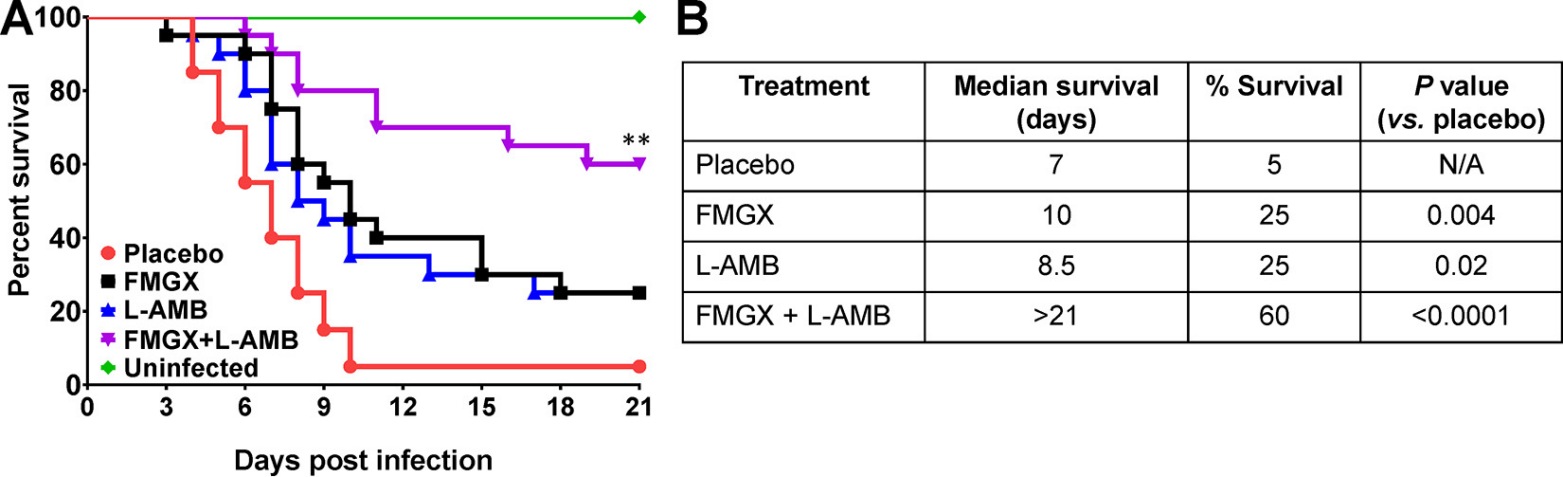
Survival of immunosuppressed mice infected with A. fumigatus. Kaplan-Meier survival curves (A) and median and percent survival values (B) show enhanced efficacy of FMGX+L-AMB combination therapy over monotherapy in protecting against murine IPA. Mice (*n* = 20/group from two independent experiments with similar results) were infected through inhalation with A. fumigatus AF293 (average inhaled inoculum of 6.7 × 10^3^ conidia/mouse) and, 48 h later, treated with FMGX (78 mg/kg) once a day (QD) PO for 7 days, L-AMB (5 mg/kg) QD intravenously (i.v.) for 4 days, or a combination of both drugs. ABT was administered 2 h prior to each antifungal treatment. **, *P* < 0.02 versus all other groups by log rank test. N/A, not applicable.

In the severe delayed-treatment IPA model, after treatment for 7 days with FMGX and 4 days with L-AMB, 60% survival was observed at day 21 for the combination therapy cohort, whereas 25%, 25%, and 5% survival were observed for the FMGX, L-AMB, and placebo cohorts, respectively (Fig. 1A). Furthermore, the median survival times were determined to be 7 days for the placebo cohort, 10 days for the FMGX cohort, 8.5 days for the L-AMB cohort, and >21 days for the FMGX+L-AMB cohort (Fig. 1B). While all treatments resulted in significant prolongations of median survival times and increased overall survival compared to the outcomes for placebo-treated mice, combination therapy significantly enhanced the survival outcomes of mice even compared to those of L-AMB or FMGX monotherapy (*P* < 0.02) (Fig. 1).

**FIG 2:**
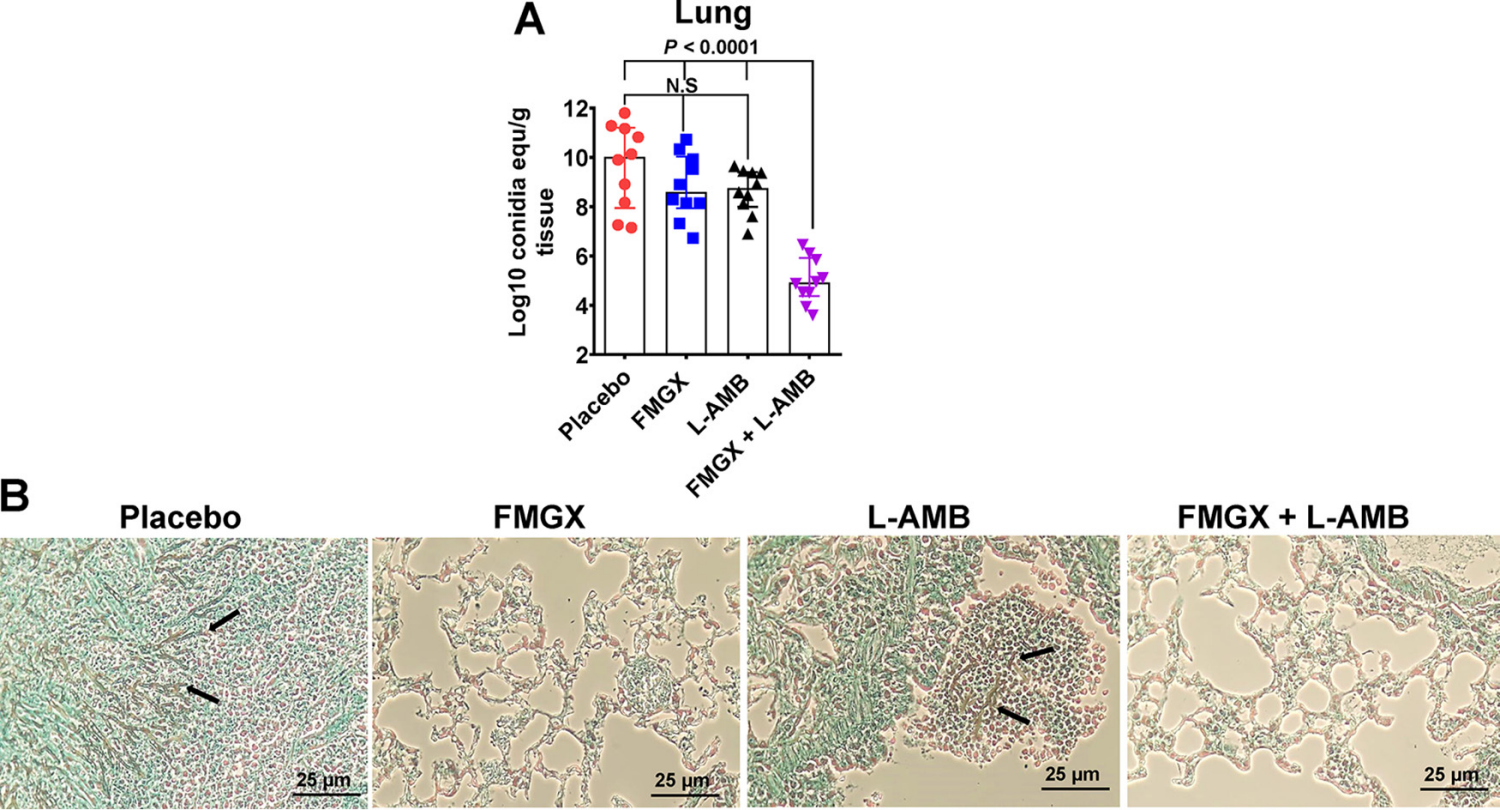
Lung fungal burdens and histopathology show enhanced efficacy of FMGX+L-AMB combination therapy in immunosuppressed mice infected with A. fumigatus. Mice (*n* = 10/group) were infected through inhalation of A. fumigatus AF293 (inhaled inoculum of 6.0 × 10^3^ conidia/mouse) and, 48 h later, treated with FMGX (78 mg/kg) QD PO, L-AMB (5 mg/kg) QD i.v., or a combination of both drugs. ABT was administered 2 h prior to each antifungal treatment. (A) On day +4, ~6 h after the third treatment, lungs were collected and processed for tissue fungal burden analysis by qPCR. Data are the median values ± interquartile ranges, and the bottom of the *y* axis represents the lower limit of detection. Only combination therapy resulted in a statistically significant reduction in lung fungal burden versus all other treatments (Wilcoxon rank sum test). equ, equivalent; N.S., not significant. (B) Histological examination of lung sections with GMS revealed focal fungal pneumonia in the placebo-treated mice (indicated by abscesses with elongated intact hyphae; arrows) and tissue edema versus smaller abscesses in the L-AMB-treated mice, which had residual fragmented fungal hyphae and less tissue edema (arrow). Treatment with either FMGX or the combination of FMGX+L-AMB resulted in normal lung architecture with no signs of fungal pneumonia.

We also evaluated the efficacy of combination therapy in reducing the tissue fungal distribution in the lungs of mice infected with A. fumigatus. Immunosuppressed mice were infected through inhalation, and treatment with either FMGX or L-AMB monotherapy or FMGX+L-AMB combination therapy was initiated 48 h postinfection and lasted for 4 days. Lung fungal burdens (determined as the Log_10_ conidial equivalent using qPCR) (27) were determined in lungs harvested from mice euthanized on day 4 postinfection (i.e., the third day of treatment, ~6 h after the last treatment). As can be seen by the results in Fig. 2A, only combination therapy resulted in a significant, ~5-Log_10_ reduction in lung fungal burden compared to the lung fungal burden in placebo-treated mice. In addition, combination treatment of FMGX+L-AMB resulted in 3- to 4-Log_10_ reductions in fungal burdens compared to either monotherapy (Fig. 2A). These results were corroborated by the histopathological examination of lungs harvested on the same day as tissue processed for quantification of the fungal burden. Specifically, while lungs harvested from placebo-treated mice showed extensive fungal hyphae surrounded by phagocytes, with signs of fungal pneumonia and a substantial degree of tissue edema, lungs from mice treated with FMGX or combination therapy showed normal tissue architecture with no apparent fungal hyphae (Fig. 2B). Lungs harvested from mice treated with L-AMB showed some fungal abscesses with lesser signs of pneumonia and tissue edema. Collectively, these data demonstrate the benefit of combination therapy of FMGX+L-AMB in treating murine IPA compared to the outcomes for placebo- and monotherapy-treated mice.

### Combination therapy of FMGX and L-AMB is superior to monotherapy in treating murine IM.

**FIG 3:**
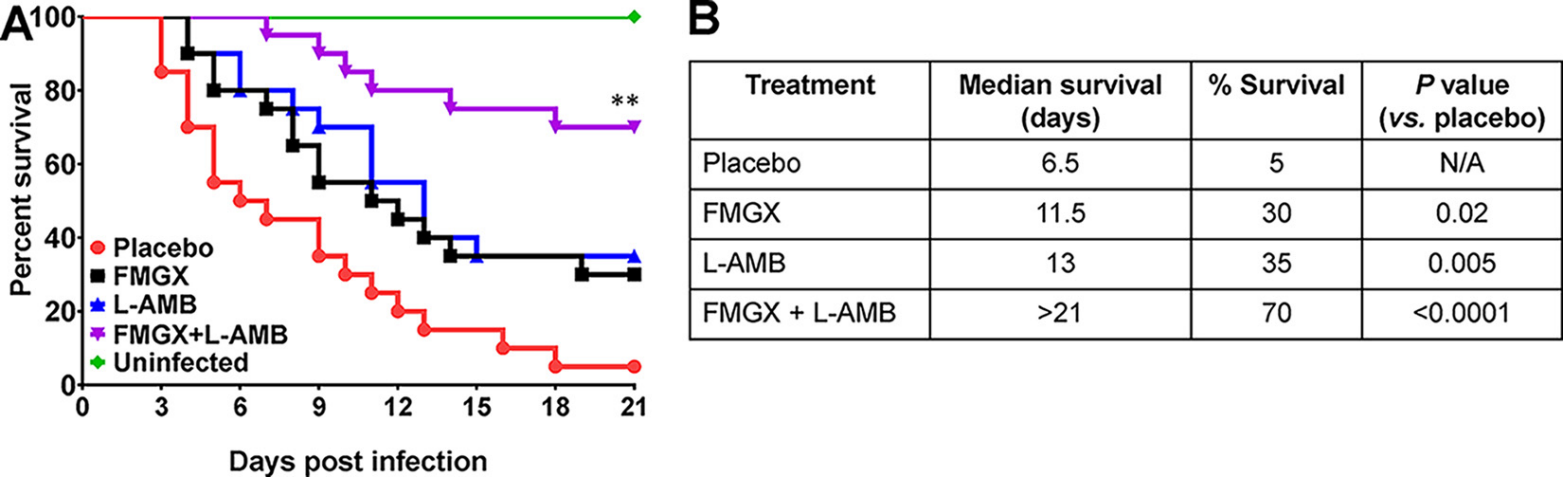
Survival of immunosuppressed mice infected with R. arrhizus var. *delemar*. Kaplan-Meier survival curves (A) and median and percent survival values (B) show enhanced efficacy of FMGX+L-AMB combination therapy over monotherapy in protecting against murine IM. Mice (*n* = 20/group from two independent experiments with similar results) were infected intratracheally with R. arrhizus var. *delemar* 99-880 (average inhaled inoculum of 2.6 × 10^4^ spores/mouse) and, 48 h later, treated with FMGX (78 mg/kg) QD PO for 7 days, L-AMB (10 mg/kg) QD i.v. for 4 days, or a combination of both drugs. ABT was administered 2 h prior to each antifungal treatment. **, *P* < 0.03 versus all other groups by log rank test. N/A, not applicable.

The efficacies of monotherapies of FMGX (78 mg/kg) and L-AMB (10 mg/kg) and of the combination of FMGX and L-AMB were evaluated versus the outcomes for the placebo vehicle control in a severe model of invasive pulmonary mucormycosis (infection with R. arrhizus var. *delemar* via intratracheal instillation) (28), where dosing was initiated 48 h postinfection. A higher dose of L-AMB was used in this severe IM model than in the IPA model. After treatment for 7 days with FMGX and 4 days with L-AMB, 70% survival was observed at day 21 for the combination therapy cohort, whereas 30%, 35%, and 5% survival were observed for FMGX-, L-AMB-, and placebo-treated mice, respectively (Fig. 3A). Moreover, the median survival times were determined to be 6.5 days for the placebo cohort, 11. 5 days for the FMGX cohort, 13 days for the L-AMB cohort, and >21 days for the FMGX+L-AMB cohort. Although all treatments resulted in significant prolongations of median survival times and increases in overall survival compared to the outcomes for placebo-treated mice, the combination therapy significantly enhanced the survival of mice even compared to either monotherapy (*P* < 0.03) (Fig. 3).

**FIG 4:**
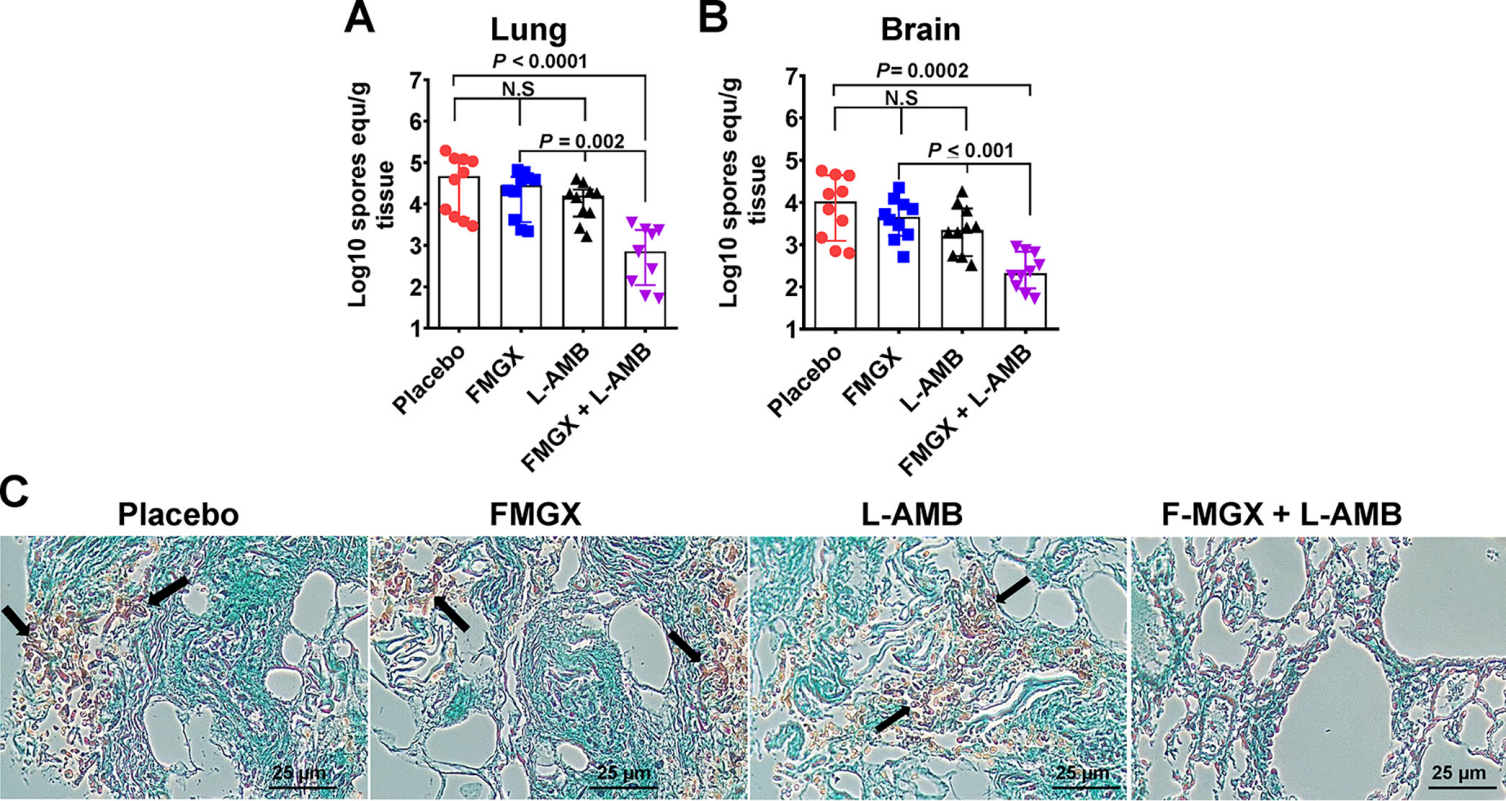
Lung and brain fungal burdens and lung histopathology show enhanced efficacy of FMGX+L-AMB combination therapy in immunosuppressed mice infected with R. arrhizus var. *delemar*. Mice (*n* = 10/group) were infected intratracheally with R. arrhizus var. *delemar* 99-880 (inhaled inoculum of 2.9 × 10^3^ spores/mouse) and, 48 h later, treated with FMGX (78 mg/kg) QD PO, L-AMB (10 mg/kg) QD i.v., or a combination of both drugs. ABT was administered 2 h prior to each antifungal treatment. (A and B) On day +4, ~6 h after the third treatment, lungs (A) and brains (B) were collected and processed for tissue fungal burden analysis by qPCR. Data are median values ± interquartile ranges, and the bottom of each *y* axis represents the lower limit of detection. Only combination therapy resulted in statistically significant reductions in tissue fungal burdens versus all other treatments (Wilcoxon rank sum test). equ, equivalent; N.S., not significant. (C) Histological examination of lung sections with GMS revealed fungal pneumonia in the placebo-, FMGX-, and L-AMB-treated mice (indicated by abscesses with broad aseptate hyphae; arrows) but not in mice treated with the combination therapy, whose lungs showed normal architecture.

Because combination therapy of FMGX+L-AMB enhanced survival over the survival in the placebo and monotherapy cohorts, we investigated the effects of this treatment regimen on the tissue fungal burdens of target organs in a separate experiment. Immunosuppressed mice were infected intratracheally with R. arrhizus var. *delemar* and treated as described above. On day 4 postinfection (i.e., ~6 h after the third treatment), mice were euthanized, lungs (primary target organ) and brains (secondary target organ) were harvested, and the fungal burdens determined as the Log_10_ spore equivalent per gram of tissue using qPCR (29). While neither of the monotherapy treatments resulted in significant reductions in tissue fungal burdens, the combination therapy of FMGX+L-AMB reduced the lung and brain fungal burdens by ~2 log compared to the burdens in placebo-treated mice. Furthermore, the combination therapy significantly reduced the fungal burdens of both organs, by ~1.0 to 1.5 log, compared to the burdens after monotherapy of either FMGX or L-AMB (Fig. 4A and B). Finally, histopathological examination of lung tissues taken from mice euthanized at the same time point as for the tissue fungal burden assessment showed that only the combination treatment resulted in resolution of fungal abscesses and of signs of pneumonia and tissue edema (Fig. 4C). Collectively, these data clearly show a benefit of the combination therapy of FMGX+L-AMB over monotherapy in treating murine IM.

### Combination therapy of FMGX and L-AMB is superior to monotherapy in treating hematogenously disseminated murine fusariosis.

**FIG 5:**
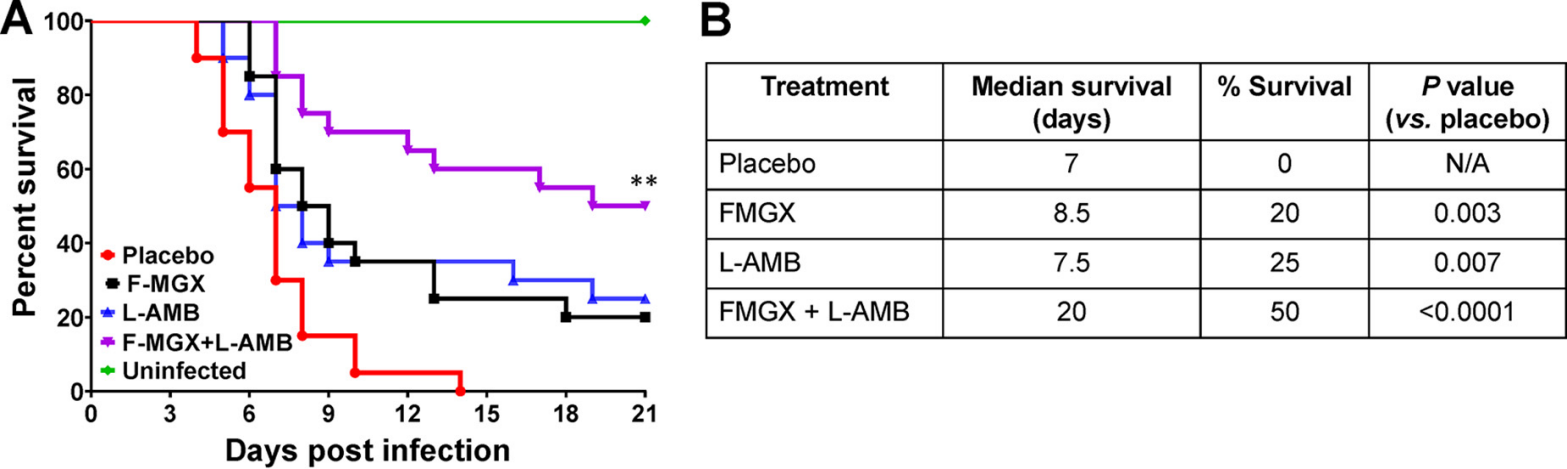
Survival of immunosuppressed mice infected with F. solani. Kaplan-Meier survival curves (A) and median and percent survival values (B) show enhanced efficacy of FMGX+L-AMB combination therapy over monotherapy in protecting against murine IF. Mice (*n* = 20/group from two independent experiments with similar results) were infected intravenously with F. solani 95-2478 (average inoculum of 8.5 × 10^2^ spores/mouse) and, 20 h later, treated with FMGX (78 mg/kg) QD PO for 7 days, L-AMB (10 mg/kg) QD i.v. for 4 days, or a combination of both drugs. ABT was administered 2 h prior to each antifungal treatment. **, *P* < 0.03 versus all other groups by log rank test. N/A, not applicable.

The efficacies of monotherapies of FMGX (78 mg/kg) and L-AMB (10 mg/kg) and of the combination of FMGX+L-AMB were evaluated versus the outcomes for the placebo vehicle control in a severe model of hematogenously disseminated fusariosis where treatment was initiated ~20 h postinfection. Similar to the IM model, a higher dose of L-AMB was used than for the IPA model. After treatment for 7 days with FMGX and 4 days with L-AMB, 50% survival was observed at day 21 for the combination therapy cohort, whereas 20%, 25%, and 0% survival were observed for FMGX-, L-AMB-, and placebo-treated mice, respectively (Fig. 5A). The median survival times were determined to be 7 days for the placebo cohort, 8.5 days for the FMGX cohort, 7.5 days for the L-AMB cohort, and 20 days for the FMGX+L-AMB cohort (Fig. 5B). Both FMGX and L-AMB monotherapies enhanced survival versus the survival in the placebo control group in this severe model (*P* = 0.003 and *P* = 0.007, respectively). Similar to what we found in murine IPA and IM, the combination of FMGX+L-AMB resulted in a significant survival enhancement versus the survival after treatment with either monotherapy (*P* ≤ 0.04) or the vehicle control (*P* < 0.0001) (Fig. 5).

**FIG 6:**
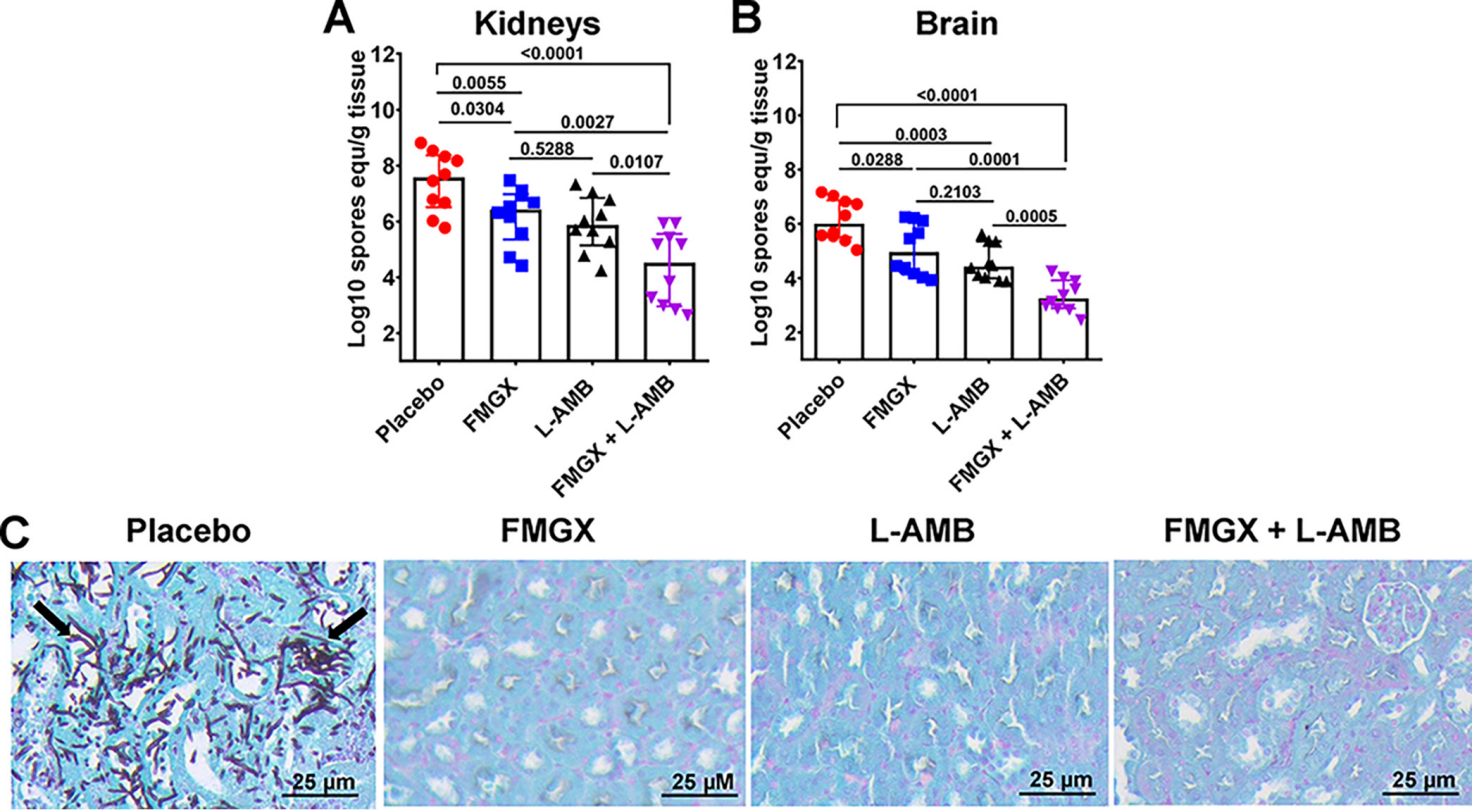
Kidney and brain fungal burdens and kidney histopathology show enhanced efficacy of FMGX+L-AMB combination therapy in immunosuppressed mice infected with F. solani. Mice (*n* = 10/group) were infected intravenously with F. solani 95-2478 (inoculum of 7.5 × 10^2^ spores/mouse) and, 20 h later, treated with FMGX (78 mg/kg) QD PO, L-AMB (10 mg/kg) QD i.v., or a combination of both drugs. ABT was administered 2 h prior to each antifungal treatment. *P* values for comparisons between treatments are shown. (A and B) On day +4, ~6 h after the third treatment, kidneys (A) and brains (B) were collected and processed for tissue fungal burden analysis by qPCR. Data are the median values ± interquartile ranges, and the bottom of each *y* axis represents the lower limit of detection. While monotherapy of either drug resulted in reduction of tissue fungal burdens versus the burdens in placebo-treated mice, the combination of FMGX+L-AMB resulted in statistically significant reductions in tissue fungal burdens versus all other treatments (Wilcoxon rank sum test). equ, equivalent. (C) Histological examination of kidney sections with GMS revealed fungal abscesses with intact fungal hyphae (arrows) in the placebo-treated mice but not in mice that received any of the other treatments.

To investigate whether the enhanced survival seen with combination therapy over monotherapy would be corroborated by the effect on tissue fungal burdens, in an independent experiment, we infected immunosuppressed mice intravenously with F. solani and treated them as described above. On day 4 postinfection (i.e., ~6 h after the third treatment), we euthanized the mice and harvested their kidneys and brains (target organs) to determine the tissue fungal burdens (using qPCR to determine the Log_10_ spore equivalent per gram of tissue [18]) and for histological examination. Both monotherapies were equally effective in lowering the kidney and brain fungal burdens, by ~1 to 2 log compared to the burdens in placebo-treated mice. Combination therapy resulted in augmented reductions in both kidney and brain fungal burdens compared to the burdens in mice treated with placebo or either monotherapy (~3-log reductions versus the burdens in mice treated with the placebo and ~1-log reductions versus mice treated with either monotherapy) (*P* ≤ 0.01) (Fig. 6A and B). Histological examination of kidneys harvested from placebo-treated mice showed significant fungal abscesses, where none of the treated mice (monotherapy or combination therapy) showed any signs of fungal infection (Fig. 6C). Collectively, these results show that combination therapy of FMGX+L-AMB is effective in treating hematogenously disseminated murine fusariosis due to F. solani and is superior to either monotherapy.

## DISCUSSION

IPA, IM, and IF are associated with high mortality rates. Several *in vitro* studies, animal models, and case series have suggested a benefit of using antifungal combination therapy over the current standard of care. For example, *in vitro* interaction between voriconazole and anidulafungin was reported to enhance antifungal activity against A. fumigatus *in vitro* (30), and case reports of patients with aspergillosis failing initial amphotericin B therapy showed a survival benefit with a combination treatment of voriconazole plus caspofungin over those treated with voriconazole alone (31). Somewhat in agreement with these findings is a randomized, double-blinded, placebo-controlled multicenter trial conducted among hematologic malignancy and hematopoietic cell transplant (HCT) patients that evaluated combination therapy of voriconazole plus anidulafungin versus voriconazole monotherapy. Although a substantial reduction in overall mortality at 6 weeks was observed in the combination arm versus the monotherapy arm (19.3% for combination therapy versus 27.5% for monotherapy), it was not significant (*P = *0.087) due to higher than expected mortality rates, which reduced the power for detection of a difference in treatment (32). Importantly, *post hoc* analysis in a subgroup of patients with probable invasive aspergillosis (based on radiographic abnormalities and galactomannan antigen positivity in the serum or bronchoalveolar lavage [BAL] fluid samples) showed a significant drop in all-cause mortality in patients treated with the combination of voriconazole plus anidulafungin versus voriconazole monotherapy (15.7% for the combination arm versus 27.3% for the monotherapy group) (*P = *0.037) (32).

*In vitro* synergy between echinocandins and polyenes was reported among Mucorales fungi and Fusarium species (33, 34). Similarly, survival benefits in mice infected with mucormycosis were also observed for echinocandins and lipid formulations of amphotericin B (35, 36). These results were corroborated by retrospective analysis conducted in two centers showing survival among rhino-orbital cerebral mucormycosis patients treated with a combination of caspofungin plus a polyene versus those treated with polyene monotherapy (37). It is noted that comparative clinical trials to assess these findings in these two diseases are challenging due to the rare nature of these two infections.

We previously showed that FMGX was as effective as antifungal drugs currently used to treat several fungal infections, including those causing IPA, IM, and IF (17, 18). Because of the high mortality rates of these three infections and the fact that, in many clinical scenarios, such infections are treated with combination therapy, we wanted to investigate whether FMGX combined with the current standard of care would enhance the survival of mice with these infections. In three animal models that mimic severely immunosuppressed patients (e.g., patients with hematologic malignancies and HCT), we showed that the combination of FMGX+L-AMB enhanced the median survival times and overall survival of mice infected with agents causing IPA, IM, and IF. These results were further confirmed by enhanced reductions in fungal burdens in target tissues and resolution of infection, as shown by histological examination, compared to the outcomes of placebo or monotherapy. However, despite the ~5-log reduction in IPA lung fungal burdens of mice treated with the combination regimen compared to the burdens in mice treated with the placebo, 40% of the mice succumbed to infection. In this model, resolution of leukopenia was associated with the onset of mouse mortality, possibly due to a hyperinflammatory response to residual infection (38).

Previous studies have shown that the combination of MGX and AMB is not synergistic *in vitro* against A. fumigatus and A. flavus (26). Although the precise mechanism by which FMGX synergistically (or additively) enhances the effect of L-AMB to treat experimental IPA, IM, and IF is yet to be determined, it is possible that inhibition of Gwt1, which results in the failure of GPI-anchored proteins to correctly localize to the outer surface structure, results in enhanced uptake of L-AMB by the microbial cell. Alternatively, attacks on the organism by two antifungal agents with two independent modes of action may result in an added microbial killing effect. Similarly, reducing the ability of the organisms to invade host tissues and evade host defenses by preventing surface proteins from localizing to the microbial cell wall could reduce their virulence, thereby enabling improved resolution of the infection. Finally, it is well documented that the immune system can recognize fungal cell surface molecules by pattern recognition receptors (PRRs) (39). It is possible that exposure of the β-glucans by the reduction of GPI-anchored localization to the outer cell wall induces better recognition by the immune cells, resulting in better clearance of infection. While cyclophosphamide treatment results in pancytopenia in mice, it has less of an effect on tissue macrophages, which are usually derived from an embryonic and not from a hematopoietic origin (40).

Our studies provide a foundation for further investigation of the benefit of combination therapy involving FMGX as the first drug in a novel group of antifungal agents. In addition to deciphering the mechanism of action and testing the benefit of combination therapy against other organisms that cause IPA, IM, and IF, combination therapy with other antifungal agents belonging to the azoles and echinocandins should be investigated. Moreover, the potential synergy/additive effects of FMGX and other antifungal agents should be evaluated against other difficult-to-treat fungal infections.

## MATERIALS AND METHODS

### Antifungal agents.

For *in vitro* studies, MGX (APX001A; Amplyx Pharmaceuticals) was used, while for efficacy studies, the water soluble *N*-phosphonooxymethyl prodrug FMGX (APX001; Amplyx Pharmaceuticals) was used. The final prodrug solution was in 5% dextrose and dosed orally (*per os* [PO]) on a per gram of mouse daily body weight basis. A 5-mg/mL solution of ABT (Fisher Scientific, Hampton, NH) in water was administered orally 2 h prior to treatment as 10 μL per gram of mouse body weight, resulting in a dose of 50 mg/kg. L-AMB was obtained from Bella Vida Pharmacy (Torrance, CA) and manufactured by Gilead Sciences (Foster City, CA) and was dosed at 5 mg/kg for IPA models or 10 mg/kg for IM and IF models.

### Microorganisms.

In this study, we used A. fumigatus strain AF293 (a generous gift of P. Magee), R. arrhizus var. *delemar* strain 99-880 (a brain isolate obtained from the Fungus Testing Laboratory at the University of Texas Health Sciences Center at San Antonio [UTHSCSA]), and F. solani strain 95-2478 (a blood isolate provided by P. Ferrieri, University of Minnesota). Routine culturing of the fungal isolates was conducted on Sabouraud dextrose agar plates at 37°C for 5 days for R. arrhizus var. *delemar* 99-880 and F. solani and for 10 days for A. fumigatus until cells were confluent. Conidia were collected by flooding the plates with sterile phosphate-buffered saline containing either 0.2% (vol/vol) Tween 80 for A. fumigatus or 0.01% (vol/vol) Tween 80 for R. arrhizus var. *delemar* 99-880 or F. solani. The conidia were concentrated by centrifugation, washed in the same buffer, diluted, and counted using a hemocytometer.

### *In vitro* testing.

The *in vitro* susceptibilities of agents of aspergillosis, mucormycosis, and fusariosis to MGX were evaluated using the Clinical Laboratory and Standards Institute (CLSI) M38-A2 method (41), using minimum effective concentration (MEC) endpoints, as for the echinocandins. For AMB, MIC endpoints were utilized.

### Efficacy models.

The pulmonary aspergillosis, pulmonary mucormycosis, and disseminated F. solani models have been previously described (16–18). For all models, CD-1 male mice weighing 20 to 23 g (Envigo) were used. Mice were immunosuppressed with cyclophosphamide (200 mg/kg) and cortisone acetate (500 mg/kg) on days −2 and +3 relative to infection for the IPA and IF models and on days −2, +3, and +8 for the IM model. To prevent bacterial infection, Baytril (50 μg/mL) was added to the drinking water from day −3 to day zero. Ceftazidime (5 μg/dose/0.2 mL) replaced Baytril treatment on day zero and was administered daily by subcutaneous injection from day zero until day +8 in the IPA and IF models and from day zero until day +13 for the IM model. To extend the half-life of MGX, mice were administered 50 mg/kg of the cytochrome P450 inhibitor 1-aminobenzotriazole (ABT) 2 h prior to FMGX administration. ABT was also added to the L-AMB treatment to control for any introduced variables. The prodrug FMGX was dosed at 78 mg/kg once daily by oral gavage to yield an actual dose of MGX of 60 mg/kg (a conversion of 1.3 to account for the methyl phosphate group in the prodrug). This dose results in exposures in mice that mimic exposures in humans achieved in phase I clinical trials using healthy volunteers (21, 22). The L-AMB doses administered via tail vein injections and used in these models included 5 mg/kg/day for IPA and 10 mg/kg/day for IM and IF.

### Infection and treatment. (i) IPA.

Immunosuppressed mice were infected with Aspergillus fumigatus in an inhalation chamber by aerosolizing 12 mL of a 1 × 10^9^ mL suspension of conidia with a small particle nebulizer driven by compressed air (38). A standard exposure time of 1 h was used for all experiments. Immediately after infection, a subset of the mice were euthanized, and the lungs were removed for quantitative culture. Treatment with FMGX (78 mg/kg/day, by oral gavage), L-AMB (5 mg/kg, administered in the tail vein), or the combination of both drugs started 48 h postinfection and continued for 7 days for FMGX and 4 days for L-AMB for survival studies. For tissue fungal burden studies, treatment started 48 h postinfection and continued for 3 days. On the third day of treatment, mice were treated in the morning and euthanized 6 h later, and target tissues were processed for fungal burden determination (see below).

### (ii) IM.

Immunosuppressed mice were challenged with Rhizopus arrhizus var. *delemar* (2.5 × 10^5^/mouse) through intratracheal instillation of 25 μL after sedation with isoflurane gas (16). Immediately after infection, a subset of mice were euthanized, lungs were removed and harvested to determine the infectious inoculum by plating on potato dextrose agar (PDA) plates containing 0.1% Triton X-100, and colonies were counted after a 24-h period at 37°C. Treatment with FMGX (78 mg/kg/day, by oral gavage), L-AMB (10 mg/kg, administered in the tail vein), or the combination of both drugs started 48 h postinfection and continued for 7 days for FMGX and 4 days for L-AMB for survival studies. For tissue fungal burden studies, treatment started 48 h postinfection and continued for 3 days. On the third day of treatment, mice were treated in the morning and euthanized 6 h later, and target tissues were processed for fungal burden determination (see below).

### (iii) IF.

Immunosuppressed mice were infected with a targeted inoculum of 8.0 × 10^2^ cells of F. solani by tail vein injection (18). Treatment with FMGX (78 mg/kg/day, by oral gavage), L-AMB (10 mg/kg, administered in the tail vein), or the combination of both drugs started ~20 h postinfection and continued for 7 days for FMGX and 4 days for L-AMB for survival studies. For tissue fungal burden studies, treatment started 48 h postinfection and continued for 3 days. On the third day of treatment, mice were treated in the morning and euthanized 6 h later, and target tissues were processed for fungal burden determination (see below).

### Tissue fungal burden studies and histopathological examination.

In all three models, we used qPCR to determine the effects of the treatments on target tissue fungal burdens, expressed as conidial equivalents, using 18S rRNA primers for IPA or IM and 28S rRNA primers for IF (17, 18, 27). Mouse DNA was detected with glyceraldehyde-3-phosphate dehydrogenase (GAPDH) primers (Table 2). Tissues harvested from mice to assess fungal burdens were also processed for histopathological examination. Briefly, tissues were fixed in 10% zinc-buffered formalin, embedded in paraffin, sectioned, and stained with Grocott’s methenamine silver (GMS) stain for microscopic examination.

**TABLE 2:** Primer sequences used for determination of tissue fungal burdens by qPCR

Model	Target^*a*^	Primer	
		Forward	Reverse
IPA	A. fumigatus 18S rRNA	GGCCCTTAAATAGCCCGGT	TGAGCCGATAGTCCCCCTAA
IM	R. arrhizus var. *delemar* 18S rRNA	GCGGATCGCATGGCC	CCATGATAGGGCAGAAAATCG
IF	F. solani 28S rRNA	TAAATGGACCAGGGCGCAAA	AGAGGGAACGAGATGGGTT
IPA, IM, and IF	Mouse GAPDH	AGGCAACTAGGATGGTGTGG	TTGATTTTGGAGGGATCTCG

a The strains used were Aspergillus fumigatus strain AF293, Rhizopus arrhizus var. *delemar* strain 99-880, and Fusarium solani 95-2478.

### Statistical analysis.

The nonparametric log rank test was used to determine differences in survival times. Differences in tissue fungal burdens were compared by the nonparametric Wilcoxon rank sum test. A *P* value of <0.05 was considered significant.

All animal-related study procedures were compliant with the Animal Welfare Act, the *Guide for the Care and Use of Laboratory Animals* (42), and the Office of Laboratory Animal Welfare, NIH, and were conducted under an IACUC-approved protocol by The Lundquist Institute for Biomedical Innovation at Harbor-UCLA Medical Center.
